# Supplementary figures and images for: Effects of extracts from various parts of invasive Solidago species on the germination and growth of native grassland plant species
Source: PeerJ. 2023 Jul 28;11:e15676. doi: 10.7717/peerj.15676 (PMC10389070; doi:10.7717/peerj.15676)

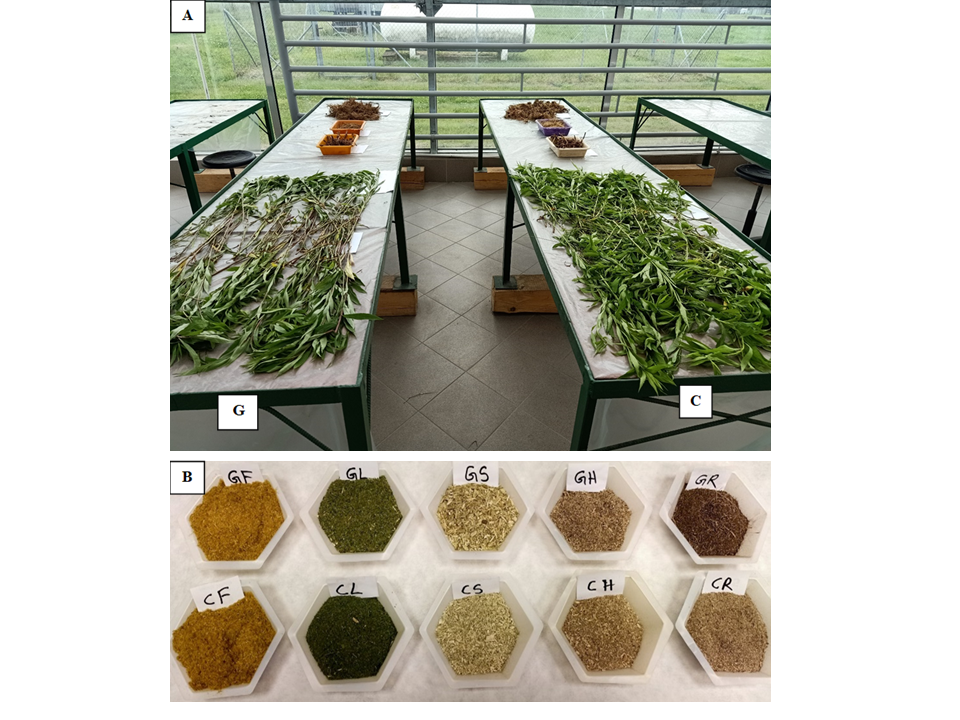

Supplement: Figure S1 [file peerj-11-15676-s001.png]

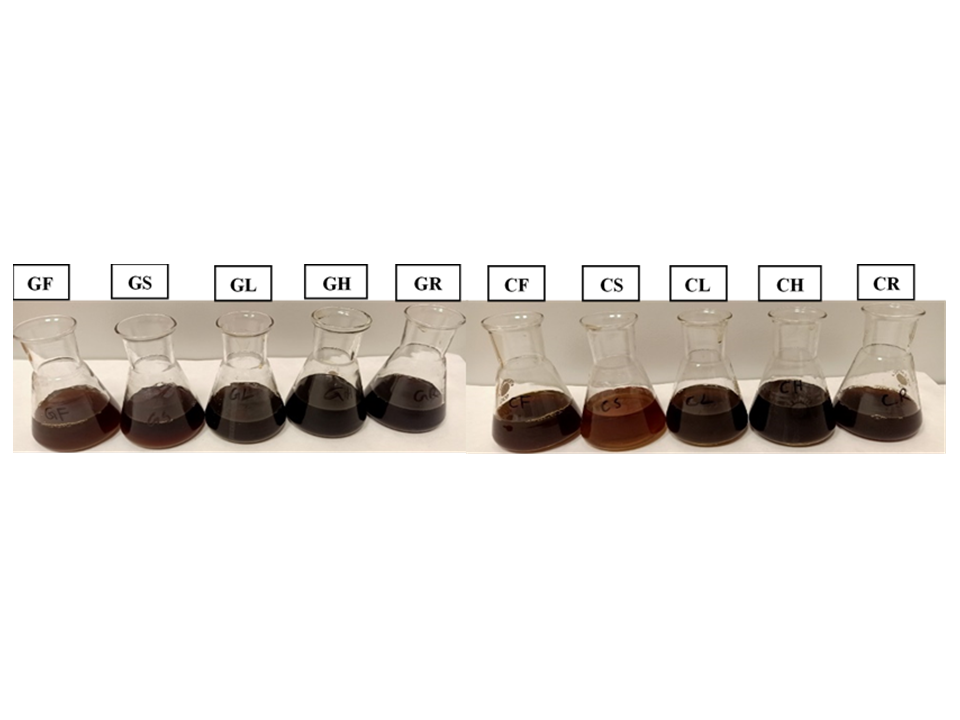

Supplement: Figure S3 [file peerj-11-15676-s002.png]

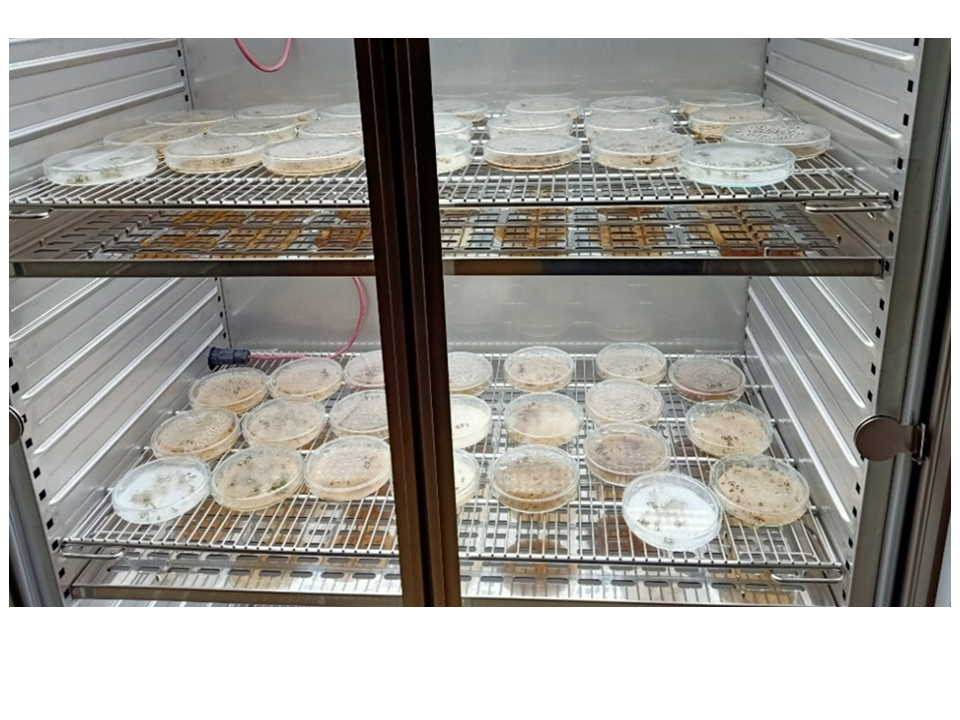

Supplement: Figure S3 [file peerj-11-15676-s003.png]

RI\_Germination

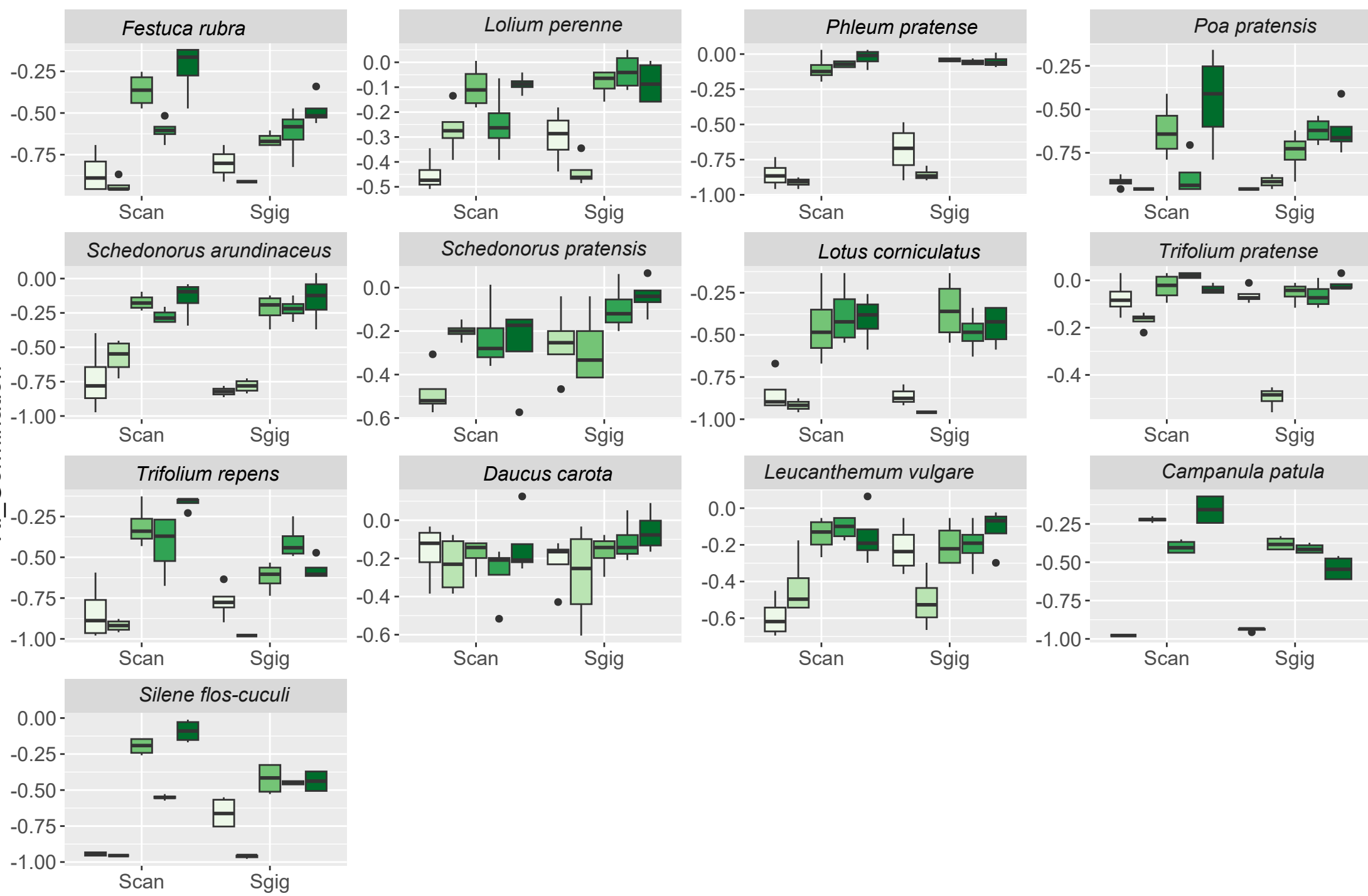

Part Flower Leaf Rhizome Root Stem

Supplement: Figure S4 [file peerj-11-15676-s004.pdf]

RI\_Root

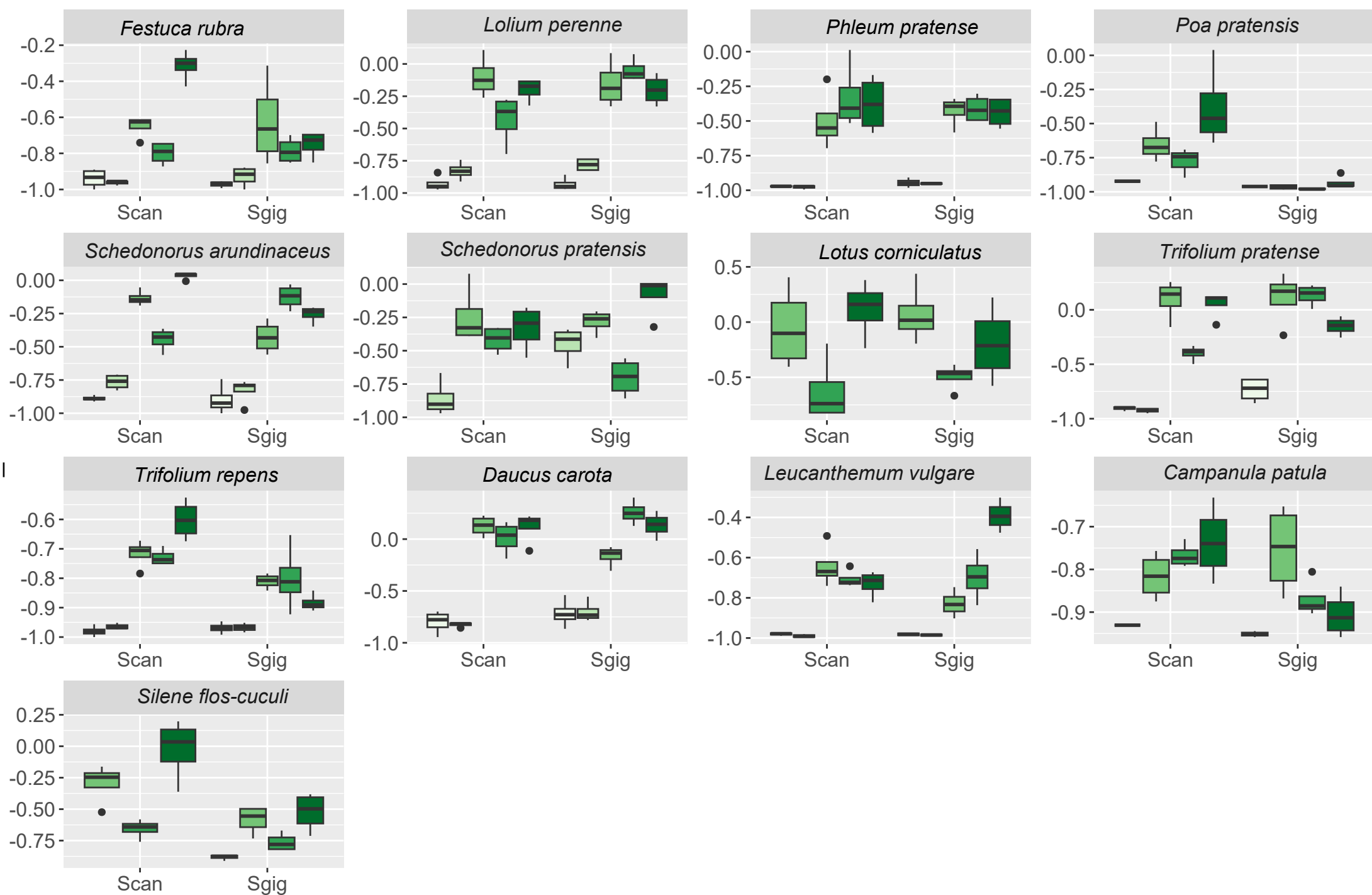

Part 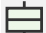 Flower 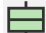 Leaf 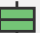 Rhizome 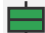 Root 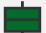 Stem

Supplement: Figure S5 [file peerj-11-15676-s005.pdf]

RI\_Shoot

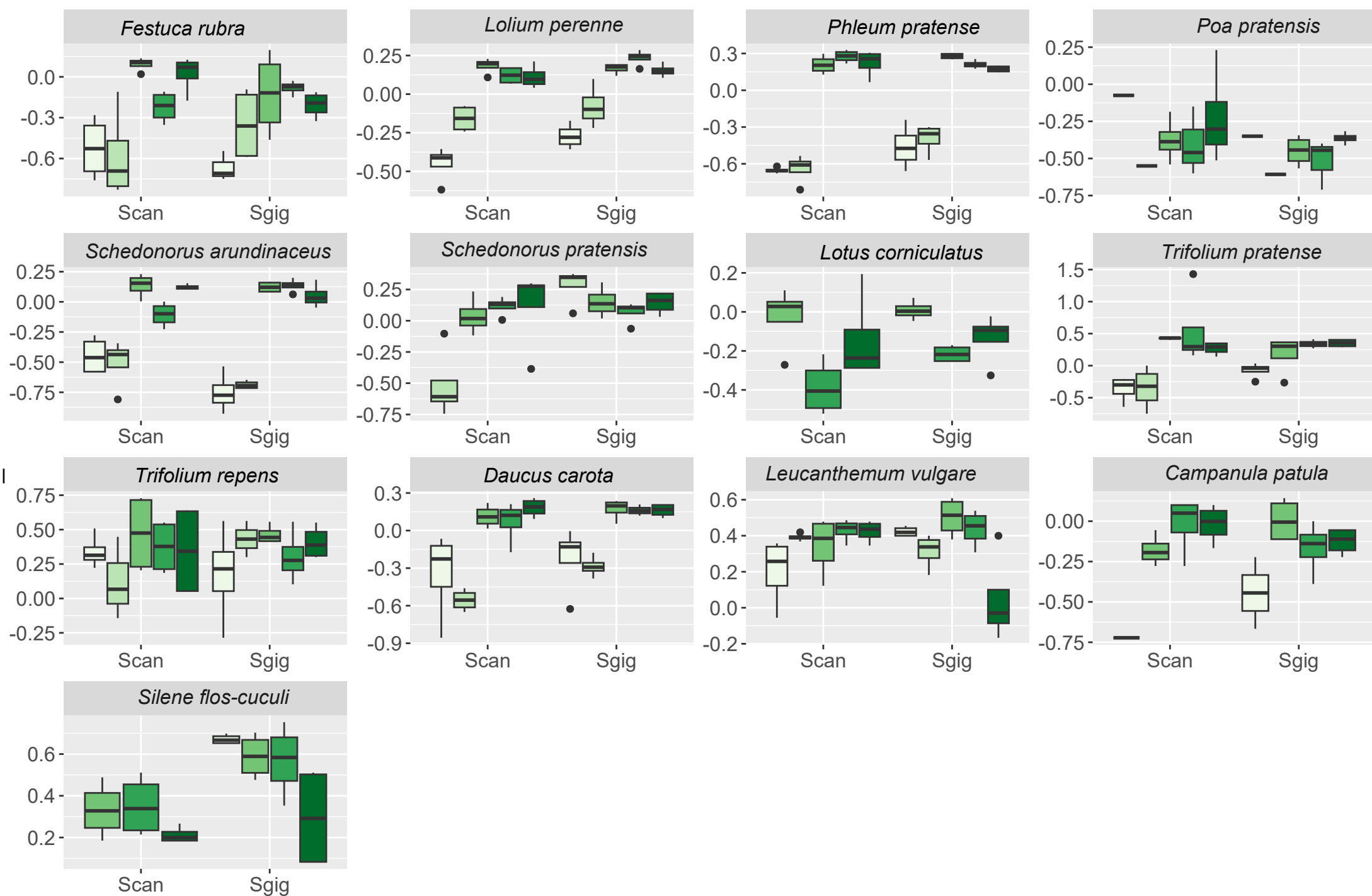

Part 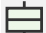 Flower 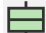 Leaf 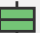 Rhizome 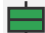 Root 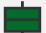 Stem

Supplement: Figure S6 [file peerj-11-15676-s006.pdf]

RI\_Weight

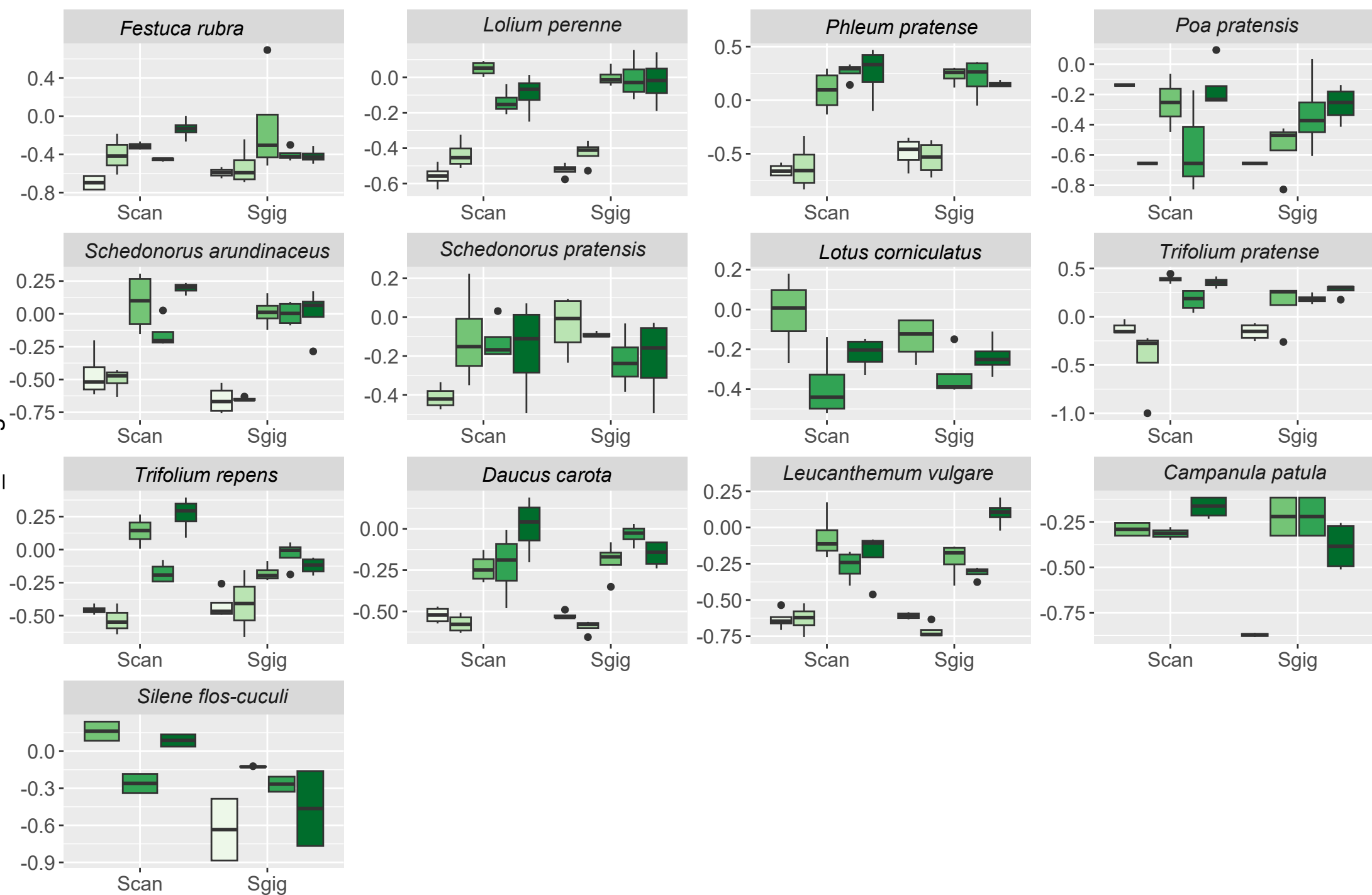

Part 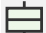 Flower 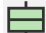 Leaf 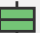 Rhizome 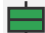 Root 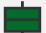 Stem

Supplement: Figure S7 [file peerj-11-15676-s007.pdf]
