# Supplementary material for: Effects of extracts from various parts of invasive Solidago species on the germination and growth of native grassland plant species
Source: PeerJ. 2023 Jul 28;11:e15676. doi: 10.7717/peerj.15676 (PMC10389070; doi:10.7717/peerj.15676)
Supplement: Table S1 — The same letters indicate a lack of significant differences (p < 0.05). [file peerj-11-15676-s008.docx]

**Supplementary Table 1:** Post hoc tests results for the response index of seed germination (RI_germ), seedlings weight (RI_weight), shoot lenght (RI_shoot), root length (RI_root) for different parts of *Solidago* (Part). The same letters indicate a lack of significant differences (p < 0.05).

| Part | RI_germ | RI_weight | RI_shoot | RI_root |
| --- | --- | --- | --- | --- |
| Flower | a | a | a | a |
| Leaf | a | a | a | a |
| Rhizome | bc | b | b | bc |
| Root | b | c | b | b |
| Stem | c | b | b | c |
