# Supplementary material for: Effects of extracts from various parts of invasive Solidago species on the germination and growth of native grassland plant species
Source: PeerJ. 2023 Jul 28;11:e15676. doi: 10.7717/peerj.15676 (PMC10389070; doi:10.7717/peerj.15676)
Supplement: Table S2 — The same letters indicate a lack of significant differences (p < 0.05). [file peerj-11-15676-s009.docx]

**Supplementary Table 2:** Post hoc tests results for the response index of seed germination (RI_germ), seedlings weight (RI_weight), shoot lenght (RI_shoot), root length (RI_root) for different grassland species (Species). The same letters indicate a lack of significant differences (p < 0.05).

| Species | RI_germ | RI_weight | RI_shoot | RI_root |
| --- | --- | --- | --- | --- |
| *Festuca rubra* | ab | a | ac | ab |
| *Lolium perenne* | de | ab | abd | cd |
| *Phleum pratense* | cd | bc | abd | abd |
| *Poa pratensis* | a | ab | c | ab |
| *Schedonorus arundinaceus* | bcd | ab | abc | cd |
| *Schedonorus pratensis* | cde | bc | bd | cd |
| *Lotus corniculatus* | ab | ab | abc | c |
| *Trifolium pratense* | e | c | de | c |
| *Trifolium repens* | ab | b | e | a |
| *Daucus carota* | de | ab | abd | c |
| *Leucanthemum vulgare* | cd | ab | e | a |
| *Campanula patula* | abc | ab | abc | ab |
| *Silene flos-cuculi* | ab | ab | e | bcd |
